# Early Adverse Events and Immune Response Following Second and Third COVID-19 Vaccination in Pregnancy

**DOI:** 10.3390/jcm11164720

**Published:** 2022-08-12

**Authors:** Shlomi Toussia-Cohen, Yoav Yinon, Ravit Peretz-Machluf, Omri Segal, Noam Regev, Keren Asraf, Ram Doolman, Yonatan Kubani, Tal Gonen, Gili Regev-Yochay, Shiran Bookstein Peretz

**Affiliations:** 1 The Department of Obstetrics and Gynecology, Chaim Sheba Medical Center, Tel-Hashomer, Ramat-Gan 52621, Israel; 2 The Sackler Faculty of Medicine, Tel-Aviv University, Tel-Aviv 69978, Israel; 3 The Dworman Automated-Mega Laboratory, Chaim Sheba Medical Center, Tel-Hashomer, Ramat-Gan 52621, Israel; 4 Infection Prevention & Control Unit, Chaim Sheba Medical Center, Tel-Hashomer, Ramat-Gan 52621, Israel

**Keywords:** adverse effects, BNT162b2 vaccine, COVID-19, pregnancy, SARS-CoV-2 IgG serum levels, third (booster) dose

## Abstract

(1) Background: The adverse-effect profile and short-term obstetric and neonatal outcomes among pregnant women who were vaccinated with the BNT162b2 vaccine at any stage of pregnancy do not indicate any safety concerns. The vaccine is effective in generating a humoral immune response in pregnant women. (2) Objective: To determine the vaccine-induced immunity and adverse events associated with the third (booster) dose of the BNT162b2 vaccine compared to the first and second dose of the vaccine among pregnant women. (3) Study design: A prospective cohort study in a tertiary referral center comparing pregnant women who were vaccinated by the first and second dose of the BNT162b2 (Pfizer/BioNTech) vaccine to pregnant women vaccinated by a third (booster) dose, between January and November 2021. A digital questionnaire regarding adverse events was filled by both groups 2–4 weeks after vaccination. Blood samples were collected and tested for SARS-COV-2 IgG antibodies 28–32 days after the administration of the second or third BNT162b2 dose. (4) Results: Seventy-eight pregnant women who received the first and second doses of the vaccine were compared to eighty-four pregnant women who received the third dose of the vaccine. In terms of adverse events following vaccination, local rash/pain/swelling (93.6% vs. 72.6%, *p* < 0.001) was significantly less common after the third vaccination compared to after the second vaccination. Other adverse events, including early obstetric complications, did not differ between the two groups. SARS-CoV-2 IgG serum levels 28–32 days after the vaccination were significantly higher after the third vaccination compared to the second vaccination (1333.75 vs. 2177.93, respectively, *p* < 0.001). (5) Conclusion: This study confirms the safety regarding early adverse events and immunogenicity, and the lack of early obstetric complications of the BNT162b2 second- and third-dose vaccine in pregnant women. The third (booster) dose is effective in generating a stronger humoral immune response in pregnant women compared with the second dose.

## 1. Introduction

Pregnant women with coronavirus disease 2019 (COVID-19) have been shown to be at increased risk for severe maternal outcomes compared to non-pregnant women, including increased risk for ICU admission, invasive ventilation, and death [1,2,3]. Despite being excluded from the initial clinical trials of the BNT162b2 vaccine and in view of the risk associated with COVID-19 in pregnancy, pregnant women in Israel were vaccinated with the first and second dose of the BNT162b2 vaccine with encouraging results regarding its safety and effectiveness in pregnancy [4,5,6,7].

Due to the emergence of the B.1.617.2 (delta) variant in Israel on July 2021 and the reduced efficacy of BNT162b2 over time, the administration of a third (booster) dose of the BNT162b2 vaccine was approved for persons who were 60 years of age or older and who had received a second dose of vaccine at least 5 months earlier, resulting in lowering rates of confirmed infection, severe illness, and COVID-19-related death [8,9,10].

Following this positive data regarding the efficacy of the booster dose, a recommendation to vaccinate high-risk populations, including pregnant women, was issued by the Israeli Ministry of Health. This recommendation was in line with the recommendations of several major international health organizations [11] and vaccination of pregnant women by the third dose was initiated. 

We recently reported [12] that the adverse-effect profile and short-term obstetric and neonatal outcomes among pregnant women who were vaccinated with the BNT162b2 vaccine at any stage of pregnancy do not indicate any safety concerns. We showed that the vaccine was effective in generating a humoral immune response of severe acute respiratory syndrome coronavirus 2 (SARS-CoV-2 IgG) levels in pregnant women [12]. Furthermore, a recent meta-analysis has compared pregnancy outcomes between vaccinated and non-vaccinated pregnant women and has shown no difference in the probability of having a small-for-gestational-age fetus and also showed reduced risk for premature delivery in vaccinated pregnant women compared with non-vaccinated pregnant women [13]. 

To date, there are no reports comparing the efficacy or safety of the third (booster) dose of the vaccine with the first and second dose among pregnant women. In this prospective cohort study, we report on the vaccine-induced immunity and adverse events associated with the third (booster) dose of the BNT162b2 vaccine among pregnant women compared with pregnant women who received the first and second dose of the vaccine.

## 2. Materials and Methods

### 2.1. Study Design and Participants 

This was a prospective cohort study comparing pregnant women who were vaccinated by the first and second dose of the BNT162b2 (Pfizer/BioNTech) vaccine to pregnant women vaccinated by a third (booster) dose, between January and November 2021. All women receiving the booster dose were fully vaccinated (i.e., had received two doses of BNT162b2) at least 5 months earlier. Women with chronic hypertension, chronic kidney disease, antiphospholipid syndrome, systemic lupus erythematosus, multiple gestation, and previous preterm birth were excluded in addition to women with positive PCR for SARS CoV-2 before or during the study period.

Participants in both groups received a digital questionnaire 2–4 weeks after the vaccine and were asked to provide information regarding demographics, medical background, history of SARS-CoV-2 infection, and side effects following vaccination.

### 2.2. Sample Collection and Processing and Antibody Quantification 

Blood samples were collected 28–32 days after the administration of the second and third dose of the BNT162b2 vaccine. Samples were centrifuged at 4000× *g* for 4 min at room temperature. Serum was tested for IgG antibodies against the SARS-CoV-2 spike RBD using the commercial automatic chemiluminescent microparticle immunoassay (CMIA) SARS-CoV-2 IgG II Quant (Abbott, Chicago, IL, USA) according to the manufacturer’s instructions.

### 2.3. Statistical Analysis

Data were described using the mean ± standard deviation (SD). Univariate analyses were performed by *t*-test for normally distributed continuous variables. The Chi-square test or Fisher’s exact test for small cell sizes were used for categorical variables. Significance was accepted at *p* ≤ 0.05. All analyses were conducted using SPSS 25 (SPSS Inc., Chicago, IL, USA).

## 3. Results

Seventy-eight pregnant women who received the first and second dose of the vaccine were compared to eighty-four pregnant women who received the third dose of the vaccine. The mean gestational age at vaccine administration in the second vaccine group was 24.23 ± 6.90 and in the third vaccine group was 21.02 ± 9.69 weeks of gestation (*p* = 0.016). The baseline characteristics of both groups are shown in Table 1. There were no significant differences between the groups with respect to age, BMI, and underlying medical conditions.

The rates of adverse events following vaccination for both groups are shown in Table 2. Local rash/pain/swelling (93.6% vs. 72.6%, *p* < 0.001) was significantly less common after the third vaccination compared to the second vaccination. Of note, none of the pregnant women in both groups experienced serious side effects such as myocarditis, anaphylaxis, or Bell’s palsy. Other adverse events, including uterine contractions, preterm premature rupture of membranes (pprom), or vaginal bleeding, did not differ between the two groups. 

The blood serology for SARS-CoV-2-specific antibodies for both groups is shown in Table 3. All serum samples in both groups were positive for SARS-CoV-2 IgG. Serum levels of SARS-CoV-2 IgG before the third vaccination were 108.01. Serum levels of SARS-CoV-2 IgG 28–32 days after the vaccination were significantly higher after the third vaccination compared to the second vaccination (2177.93 vs. 1333.75, respectively, *p* < 0.001). 

## 4. Discussion

### 4.1. Principal Findings

In this study, we examined the safety and immunogenicity of the Pfizer/BioNTech BNT162b2 third (booster) dose of the BNT162b2 vaccine among pregnant women compared with pregnant women who received the first and second dose of the vaccine.

Our results show that there were no additional adverse effects, including serious adverse effects, for the third dose of vaccination in pregnant women compared with pregnant women who received the second dose of the vaccine. Moreover, local rash/pain/swelling were significantly less common in the third dose group. None of the pregnant women included in our study experienced obstetric complications following vaccination such as uterine contractions, PPROM, and vaginal bleeding. This study showed that the first and second BNT162b2 vaccine induced humoral immunity in all vaccinated pregnant women. The serum levels of SARS-CoV-2 IgG antibodies before administration of the third dose were low. The third dose of BNT162b2 vaccine induced humoral immunity in all vaccinated pregnant women in our study, with SARS-CoV-2 IgG serum levels significantly higher after the third vaccination compared to the second vaccination. 

### 4.2. Results in the Context of What Is Known

Our results are in line with several recent studies that have shown the efficacy and safety in pregnancy of the two-dose regimen of the BNT162b2 COVID-19 vaccine [4,5,6,7,14,15], including our recent report [12] regarding the BNT162b2 booster dose. These studies have shown that vaccine-induced antibody titers were equivalent in pregnant compared with nonpregnant women and were higher than those induced by SARS-CoV-2 IgG infection during pregnancy. Our results indicate that the adverse events profile in pregnant women remains favorable with repeated doses of the vaccine. Furthermore, the risk of early obstetric side effects such as vaginal bleeding or uterine contractions remains negligible and similar to previous publications regarding the first and second doses of the vaccine in pregnancy [5].

Our finding of significantly lower rates of local rash/pain/swelling in the third vaccine group contrasts with findings in other studies that compared adverse effects in the second versus third dose of vaccine. These studies have reported similar rates of all adverse effects after the second and third vaccine doses, both in the general population and among pregnant women [16,17].

Among the group of pregnant women who received the third vaccine, we demonstrated low levels of serum SARS-CoV-2 IgG antibodies before vaccination, implicating the reduced efficacy of BNT162b2 over time as shown in several recent studies [18,19]. 

When comparing IgG serum levels after the vaccination, our results show significantly higher levels following the third vaccination compared to following the second vaccination. This finding is in accordance with a large-scale study conducted in the general population [20]. Of note, the time interval between vaccination to serology testing differed between the two groups (Table 3) and was 28.47(±3.57) and 31.94(±4.34) days for the second dose and third dose groups, respectively. However, the difference in serum IgG levels between the two groups is unlikely to be explained by this 3-day difference in blood sampling. 

### 4.3. Clinical Implications

Our results show that the third dose of the BNT162b2 vaccine is safe and effective in pregnant women and re-generates humoral immunity following the waning effect of serum IgG levels after the second dose of the vaccine. 

### 4.4. Research Implications

This study confirms the safety and immunogenicity, and the lack of early obstetric complications of the Pfizer/BioNTech BNT162b2 third dose (booster) vaccine in pregnant women. The data provided in the current study may assist health organizations to provide evidence-based recommendations regarding administration of the third dose of the COVID-19 vaccine to pregnant women. Such recommendations are especially important considering a low acceptance rate for vaccination in pregnancy. A recent systematic review of 15 studies has shown that only 49% of pregnant women are willing to be vaccinated during pregnancy [21]. However, large trials evaluating the clinical efficacy of the third dose of the vaccine in pregnancy are still needed. Furthermore, with the emergence of a fourth vaccine dose, more studies are needed to assess the safety and efficacy of repeated vaccine doses in pregnant women. 

### 4.5. Strengths and Limitations

This is the first study comparing the safety and immunogenicity of the third dose of the BNT162b2 vaccine compared to the second dose in pregnancy. Research groups were well-matched, and all of the serology blood samples were processed at the same laboratory, preventing data mismatch and bias. 

This study is limited by its relatively short time frame, limiting our ability to observe possible longer-term adverse events and pregnancy outcomes, and to assess the clinical effectiveness of the vaccine. Another limitation is our use of questionnaires, which could lead to respondent bias.

## 5. Conclusions

This study confirms the safety regarding early adverse events and immunogenicity, and the lack of early obstetric complications of the BNT162b2 s and third dose vaccine in pregnant women. The third (booster) dose is effective in generating a stronger humoral immune response in pregnant women compared with the second dose.

## Figures and Tables

**Table 1:** Baseline characteristics of 78 pregnant women who received a second dose and 84 pregnant women who received a third (booster) dose of BNT162b2 vaccine.

Characteristic	Second Vaccination (n = 78)	Third Vaccination (n = 84)	*p* Value
Age	32.85 (± 3.49)	33.23(±3.95)	0.519
Mean gestational age at vaccine administration (weeks)	24.23 (±6.90)	21.02 (±9.69)	0.016
BMI	24.74 (±5.43)	23.41 (±4.137)	0.79
Autoimmune Disease	18 (23.1%)	14 (16.7%)	0.306
Lung Disease	2 (2.6%)	3 (3.6%)	0.711
Diabetes	2 (2.6%)	0 (0%)	0.14
Cardiovascular Disease	0 (0%)	3 (3.6%)	0.92

Data are given as mean ± SD or n (%). BMI, body mass index.

**Table 2:** Adverse events after BNT162b2 vaccine in 78 pregnant women who received a second dose and 84 pregnant women who received a third (booster) dose.

Adverse Event	Second Vaccination (n = 78)	Third Vaccination (n = 84)	*p* Value
Rash/Local pain/Local swelling	73 (93.6%)	61 (72.6%)	<0.001
Gastrointestinal symptoms	13 (16.7%)	8 (9.5%)	0.176
Fever (37.5 and up)	7 (9.0%)	8 (9.5%)	0.579
Weakness and fatigue	45 (57.7%)	32 (38.1%)	0.013
Myalgia	21 (26.9%)	17 (20.2%)	0.316
Axillary lymphadenopathy	2 (2.6%)	6 (7.1%)	0.179
Remote lymphadenopathy	3 (3.8%)	7 (8.3%)	0.236
Paresthesia	4 (5.1%)	4 (4.8%)	0.914
Headache	5 (6.4%)	3 (3.6%)	0.405
Bell’s palsy	0 (0%)	0 (0%)	N/A
Myocarditis	0 (0%)	0 (0%)	N/A
Anaphylaxis	0 (0%)	0 (0%)	N/A
Hospitalization	0 (0%)	0 (0%)	N/A
Uterine Contractions	1 (1.3%)	3 (3.6%)	0.348
Vaginal Bleeding	0 (0%)	0 (0%)	N/A
PPROM	0 (0%)	0 (0%)	N/A

Data are given as n (%). N/A, not applicable. PPROM—preterm premature rapture of membranes.

**Table 3:** Blood serology for SARS-CoV-2-specific antibodies in in 78 pregnant women who received a second dose and 84 pregnant women who received a third (booster) dose of BNT162b2 vaccine.

Variable	Second Vaccination (n = 78)	Third Vaccination (n = 84)	*p* Value
Serum IgG Before third dose (BAU/mL)	N/A	108.01 (±74.69)	
Time from vaccination to serology test (days)	28.47 (±3.57)	31.94 (±4.34)	<0.001
Serum IgG 30 days after third dose (BAU/mL)	1333.75 (±917.35)	2177.93 (±1525.89)	<0.001

Data are given as mean ± SD or median (interquartile range). IgG, immunoglobulin G. BAU/mL, binding antibody units per milliliter.

## Data Availability

The data presented in this study are available on request from the corresponding author.
